# Phylogeny and taxonomy of *Iris* (Iridaceae) series *Tenuifoliae*: Discovery of a clade with chloroplast genome inversion and description of three new species

**DOI:** 10.3897/phytokeys.275.184036

**Published:** 2026-06-02

**Authors:** Zhongzheng Zhang, Huatian Li, Gexiang Zhang, Taoyi Zuo

**Affiliations:** 1 College of Landscape Architecture, Nanjing Forestry University, Nanjing 210037, China College of Landscape Architecture, Nanjing Forestry University Nanjing China; 2 Beijing, China Unaffiliated Beijing China

**Keywords:** Chloroplast genome, inversion region, Iris series Tenuifoliae, new species, phylogeny

## Abstract

*Iris* series *Tenuifoliae* (Diels) G.H.M.Lawr., which is widely distributed across Central and East Asia, comprises approximately 10 recognized taxa. However, taxonomic boundaries and phylogenetic relationships within the series have remained ambiguous due to considerable morphological complexity and insufficient molecular evidence. To resolve this, we conducted an integrated study using complete chloroplast genome sequences and morphological data. Our results clearly delineate the series’s boundaries and establish its phylogenetic position within the genus *Iris*. The series was resolved into three strongly supported clades, with the circumscription of one clade corroborated by a specific inversion in the Small Single-Copy (SSC) region of the chloroplast genome. Furthermore, we describe 3 new species from central-western China: *Iris liupanshanensis* H.T.Li, Z.Z.Zhang & T.Y.Zuo, **sp. nov**., *Iris helanshanensis* Z.Z.Zhang, T.Y.Zuo & H.T.Li, **sp. nov**. and *Iris coeruleoviridis* T.Y.Zuo, H.T.Li & Z.Z.Zhang, **sp. nov**.

## Introduction

The genus *Iris* L. (*s.l*.) is a member of the tribe Irideae Kitt. in the family Iridaceae Juss., comprising approximately 300 species (Zhao 1985; Goldblatt and Manning 2008). The generic name is derived from Ίρις, the goddess of the rainbow in Greek mythology. Linnaeus (1753) formally adopted and published this name for the genus in *Species Plantarum*.

This genus is widely distributed across temperate and subtropical regions of the Northern Hemisphere, exhibiting significant morphological variation (Crespo et al. 2015). A particular group originating from Central and East Asia possesses a series of distinctive diagnostic characteristics. Plants of this group are tufted, with persistent fibrous leaf sheaths—an evolutionary adaptation to arid environments (Rodionenko 2006). The roots are tough and scarcely branched, developing from either prominent or inconspicuous rhizomes. The flowering stem is typically unbranched and often so short that it does not emerge above the ground. Flowers display the characteristic floral morphology of *Iris*. Notably, the ovary of this group features a slender beak, a characteristic also observed in species such as *I. unguicularis* Poir., *I. sintenisii* Janka and *I. macrosiphon* Torr. (Akyol et al. 2014). The capsule usually has a short or long beak, and the seeds lack appendages (Dykes 1912; Mathew 1989).

Baker (1876) was the first to recognize this taxon, which originally included *I. tenuifolia*, *I. ventricosa* Pall., *I. songarica* Schrenk ex Fisch. & C.A.Mey., and *I. macrosiphon*. Dykes (1912), while removing *I. macrosiphon* and *I. songarica*, retained *I. tenuifolia* and *I. ventricosa* within the group and incorporated *I. bungei* Maxim., which had been described by Maximowicz (1880). Subsequent revisions of this group have largely followed the frameworks established by Baker (1876) and Dykes (1912). Diels (1930) formally named this taxon *I.* sect. *Apogon* Baker subsect. *Tenuifoliae* Diels. Rodionenko (1961) further subdivided *I.* subsect. *Tenuifoliae* into 2 series: *I.* ser. *Tenuifoliae* (Diels) G.H.M.Lawr., characterized by a very short, subterranean flowering stem, and *I.* ser. *Ventricosae* Rodion., defined by its distinct, above-ground aerial stem. Mathew (1989) only recognized *I.* ser. *Tenuifoliae* within *I.* sect. *Limniris* Tausch to encompass these taxa without further subdivision. Under the circumscription of Mathew (1989), this series comprises a total of 10 species, namely *I. bungei*, *I. cathayensis*, *I. kobayashii* Kitag., *I. loczyi* Kanitz, *I. songarica*, *I. tenuifolia*, *I. ventricosa*, *I. anguifuga* Y.T.Zhao & X.J.Xue, and *I. qinghainica* Y.T.Zhao, *I. polysticta* Diels (now considered a synonym of *I. farreri* Dykes).

However, due to considerable morphological and ecological variation among these species, the monophyly of this series has been widely questioned. One of these species is *I. anguifuga*, from east-central China. Upon describing the species, Zhao (1980), citing its unique morphological traits, therefore proposed *I.* sect. *Ophioiris* Y.T.Zhao for its placement. Rodionenko (2004) later elevated it to the genus level as *Ophioiris* (Y.T.Zhao) Rodion. Crespo et al. (2015) integrated existing phylogenetic analyses and morphological characteristics, placing it within *Chamaeiris* Medik.—corresponding to *I.* ser. *Foetidissimae* (Diels) B.Mathew and *I.* ser. *Spuriae* (Diels) G.H.M.Lawr.—though its phylogenetic position within the genus has not been fully resolved. Another species that has raised taxonomic uncertainty is *I. songarica*, which produces a long scape bearing 2–3 flowers and is sometimes branched, with 2–4 branches (Boltenkov and Schröder 2019). Nevski (1937) proposed the genus *Sclerosiphon* Nevski to accommodate this morphologically distinct species. Rodionenko (2006) further expanded the scope of *Sclerosiphon*, including *I. ventricosa* and *I. bungei* in this genus based on stable and consistent morphological and anatomical characteristics. Wilson (2009) resolved *I. songarica* as the sister group to *I. lactea* Pall. The analysis by Mavrodiev et al. (2014) and Crespo et al. (2025) yielded a congruent result; while advocating for the reinstatement of *Sclerosiphon*, they retained *I. ventricosa* and *I. bungei* within *I.* ser. *Tenuifoliae* (which they proposed to treat at the generic level as *Cryptobasis* Nevski).

Regarding the phylogenetic position of *I.* ser. *Tenuifoliae* within the genus *Iris*, different studies have yielded conflicting conclusions. The results of Makarevitch et al. (2003), based on a phylogeny constructed from the *trnL* intron and *trnL-trnF* intergenic spacer, strongly supported a sister-group relationship between *I.* ser. *Spuriae* and *I.* ser. *Tenuifoliae*. Wilson (2004) recovered a clade comprising *I. loczyi* and *I. missouriensis* Nutt. as the sister group to a clade containing *I.* ser. *Spuriae* and I. subg. Xiphium (Mill.) Spach, but this relationship had low statistical support. In the study by Mavrodiev et al. (2014), based on conventional phylogenetic analyses, *I.* ser. *Tenuifoliae (Cryptobasis)* was found to be closely related to taxa such as I. subg. Hermodactyloides Spach (*Iridodictyum* Rodion.), *I.* ser. *Syriacae* (Diels) G.H.M.Lawr. (*Syrianthus* M.B.Crespo, Mart.-Azorín & Mavrodiev), *Hermodactylus* Mill., I. subg. Xiphion Mill.), *I.* ser. *Foetidissimae*, and *I.* ser. *Spuriae* (both as *Chamaeiris* Medik.); however, its internal topology was unstable.

This study intends to utilize complete chloroplast genome data combined with morphological comparative analyses to address the following issues: (1) clarify the delimitation of *I.* ser. *Tenuifoliae* and its phylogenetic position within the genus *Iris*; (2) analyze structural variation characteristics such as IR boundary expansion/contraction and inversions in the chloroplast genomes of *I.* ser. *Tenuifoliae*; (3) reveal the major evolutionary lineages within *I.* ser. *Tenuifoliae* and explore their distribution patterns; and (4) provide formal descriptions of 3 taxa. This work aims to contribute to refining the taxonomy of the genus *Iris* and enhance the understanding of its evolutionary history.

## Materials and methods

### Morphological sampling and examination

In mid-June 2025 (flowering period) and late August 2025 (fruiting period), detailed field investigations and morphological observations were conducted on 3 putative new taxa. During this process, 2 unidentified taxa were collected and provisionally designated as *I.* sp. A and *I.* sp. B, which are characterized by the absence of a conspicuous aerial stem. To avoid confusion, it is important to clarify that these 2 unidentified taxa do not fall within the scope of the 3 new species described in this study. The herbarium specimens were sourced from platforms including the Chinese Virtual Herbarium (CVH) (https://www.cvh.ac.cn/), the Global Biodiversity Information Facility (https://www.gbif.org/), and Integrated Digitized Biocollections (https://www.idigbio.org/). These specimens, representing all known taxa of *I.* ser. *Tenuifoliae*, are deposited in herbaria such as A, BNU, CDBI, E, GH, HBNU, HIB, HNWP, IATM, IBSC, IFP, IRKU, JLSLKY, K, KUN, LD, LZD, MICH, MW, MU, MNHN, NAS, NEFI, NMU, NWTC, PE, PEY, RSA, S, TIE, US, and ZMNH. Specific details of the examined specimens for each species, along with their collection codes, are provided in Suppl. material 1.

### Molecular sampling, DNA sequencing, and plastid genome assembly

Fresh leaf samples were collected from both wild and cultivated individuals, yielding a total of 21 specimens. Among these, 4 samples (representing 4 species) belonged to *I.* ser. *Spuriae*, while the remainder were from *I.* ser. *Tenuifoliae*, including 3 newly described species and 2 unidentified taxa. Detailed procedures for DNA extraction, quality assessment, library preparation, sequencing, and raw data quality control follow those described in a previous study (Zhang and Zhang 2026). High-quality reads after quality control were used for plastome assembly with GetOrganelle v1.7.7.1 (Jin et al. 2020). The assembly results were annotated using CPGAVAS2 (Shi et al. 2019), with *I. loczyi* (MT254070.1) as the reference genome. Subsequently, manual verification and adjustment of the annotations were performed using CPStools (Huang et al. 2024). The newly assembled plastome sequences generated in this study were deposited in the GenBank database; specific accession numbers are provided in Suppl. material 2. Circular genome maps were drawn using OGDRAW (Lohse et al. 2007).

### Interspecific genome comparative analyses

Comparative alignment among plastomes and the detection of plastome rearrangement events were performed using Mauve v2.4.0 (Darling et al. 2004). Visualization and analysis of the LSC, SSC, and IR boundary regions in the chloroplast genomes of *I.* ser. *Tenuifoliae* were conducted with CPJSdraw (Li et al. 2023) to investigate IR expansion and contraction.

### Phylogenetic analyses

In the phylogenetic reconstruction of the *Iris* genus, a total of 70 chloroplast genomes were collected from 63 taxa. Among these, 21 samples were newly sequenced in this study; 5 were obtained from previous research; 13 partial chloroplast genomes were derived from the study by Wilson et al. (2023); and the remainder were sourced from GenBank (see Suppl. material 2). Following earlier phylogenetic studies on Iridaceae (Kamra et al. 2023), the chloroplast genomes of *Moraea spathulata* (L.f.) Klatt and *M. polystachya* (Thunb.) Ker Gawl. were used as outgroups.

To address the IRa inversions observed in some chloroplast genomes, the inverted fragments were manually reverse-complemented by aligning them with a reference sequence. Subsequently, the LSC, IRb, and SSC regions were uniformly extracted from each sequence to construct the final data matrix. Multiple sequence alignment of the above data matrix was performed using MAFFT v7.525 (Katoh and Standley 2013) and subsequently trimmed using trimAl (Capella-Gutiérrez et al. 2009). The best-fit substitution model was determined based on the Bayesian Information Criterion (BIC) using ModelFinder ver. 2.2.0 (Kalyaanamoorthy et al. 2017). The Maximum Likelihood (ML) phylogenetic tree was reconstructed using IQ-Tree v.3.0.1 (Nguyen et al. 2015) under the K3Pu+F+R4 model, using 1000 ultrafast bootstrap replicates to estimate nodal support. In addition, Bayesian Inference was conducted using MrBayes v.3.22 (Ronquist et al. 2012). 2 independent Markov Chain Monte Carlo (MCMC) runs were executed, each with 5 million generations, sampling every 1000 generations. The first 25% of samples were discarded as burn-in to ensure the convergence and stability of the posterior probability distribution.

## Results

### Morphological comparison

In *I.* ser. *Tenuifoliae*, *I. liupanshanensis* sp. nov. is particularly distinctive for its leaves reaching up to 1.2 cm in width and its firm texture, with inconspicuous longitudinal veins. *I. liupanshanensis*, *I. ventricosa*, *I. bungei*, and *I. songarica* all possess prominent above-ground stems; specific distinctions among them are presented in Table 1. The most distinctive characteristic of *I. helanshanensis* sp. nov. within *I.* ser. *Tenuifoliae* is the absence of an elongated ovary neck. The overall morphology of *I. helanshanensis* resembles that of *I. farreri*, reflected in the indistinctly woody, tuberous rhizomes and the deep bluish-purple flowers (differences are summarized in Table 2). *I. coeruleoviridis* sp. nov. is morphologically similar to *I. anguifuga*, sharing solitary flowers, fruits with long beaks, and stout, fleshy rhizomes. However, they differ in flower size, leaf width, and bract length (Table 3).

**Table 1.** Main morphological differences between *Iris liupanshanensis*, *I. bungei*, *I. ventricosa*, and *I. songarica*.

Character	*I. liupanshanensis*	*I. bungei*	*I. ventricosa*	*I. songarica*
Leaf width	10–12 mm	2–4 mm	3–4 mm	3–6 mm
Plant height	25–40 cm	15–25 cm	10–15 cm	40–80 cm
Bract shape	Broadly lanceolate	Broadly ovate to ovate	Ovoid to broadly lanceolate	Broadly lanceolate
Bract length	10–13 cm	8–10 cm	6–8 cm	9–12 cm
Fruit	Long-ovoid	Cylindrical-narrowly ovoid	Trigonous-ovoid	Oblong

**Table 2.** Main morphological differences between *Iris helanshanensis* and *I. farreri*.

Character	*I. helanshanensis*	*I. farreri*
Leaf width	4–6 mm	4–12 mm
Bract	6.4–7 × 0.5–1 cm	7–14 × 1.8–2 cm
Flowers per stem	2 flowers	1–2 flowers
Perianth tube	Cyathiform	Funnelform
Ovary	Trigonous-ovoid, 1.4–2 cm long, neck absent	Trigonous-fusiform, 2.3–2.5 cm long, including a neck ca. 1.30 cm long
Style branches	Unspotted	Densely covered with deep purple spots at base

**Table 3.** Main morphological differences between *Iris coeruleoviridis* and *I. anguifuga*.

Character	*I. coeruleoviridis*	*I. anguifuga*
Leaf width	7–10 (12) mm	5–7 mm
Bract length	6–9.5 cm	10–13.5 cm
Number of bracts	2	1
Number of stem bract-like leaves	1	2
Flower diameter	6–6.5 cm	ca. 10 cm
Fruit size	6.5–8 cm long, ca. 1.5 cm in diameter	5.5–7 cm long, 1.5–2 cm in diameter

It is worth noting that dried specimens exhibited significantly narrower leaf widths than fresh leaves due to shrinkage, a factor that should be considered in species identification.

### General chloroplast genome features

**Figure 1.**
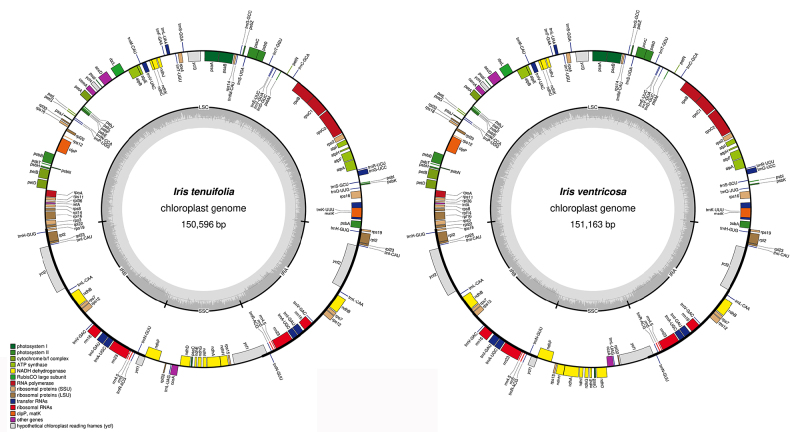
Complete chloroplast genome maps of *Iris tenuifolia* and *I. ventricosa*. The gray bars in the inner circle indicate the GC content. Genes belonging to different functional groups are color-coded.

The complete chloroplast genomes of the 21 *Iris* samples obtained in this study ranged in size from 150,596 bp (*I. tenuifolia*) to 157,200 bp (*I. anguifuga* 2) (Suppl. material 2). All 21 plastomes exhibit the typical quadripartite structure, consisting of 2 inverted repeat (IR) regions, a large single-copy (LSC) region, and a small single-copy (SSC) region (Fig. 1), with lengths of 25,526–30,065 bp, 81,249–83,640 bp, and 13,430–18,150 bp, respectively. The chloroplast genomes of all 21 *Iris* samples contained 113 genes, comprising 79 protein-coding genes, 30 tRNA genes, and 4 rRNA genes (Suppl. material 3).

### Comparative analyses

**Figure 2.**
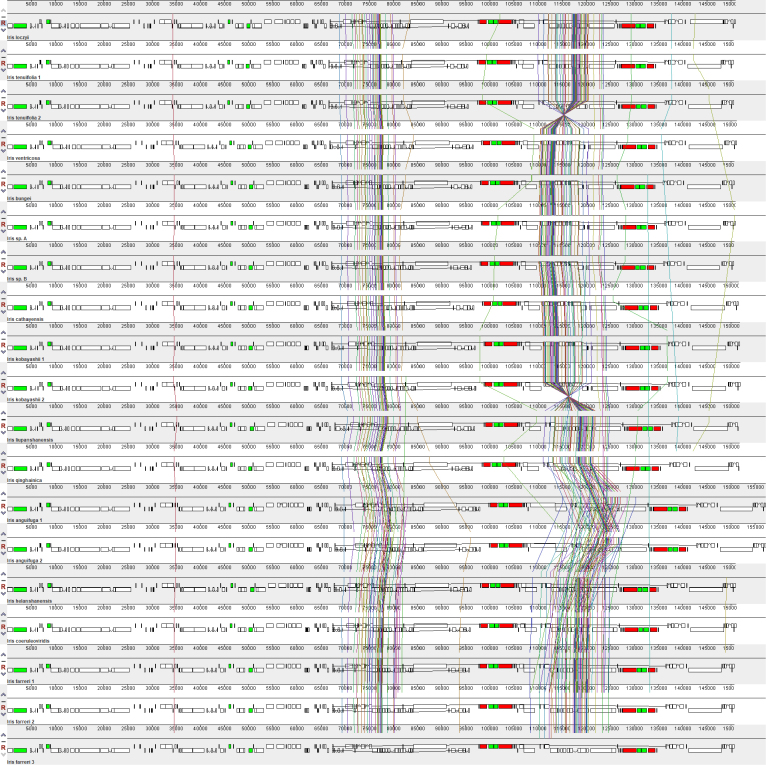
Synteny and rearrangements detected in the plastomes of *I.* ser. *Tenuifoliae*.

**Figure 3.**
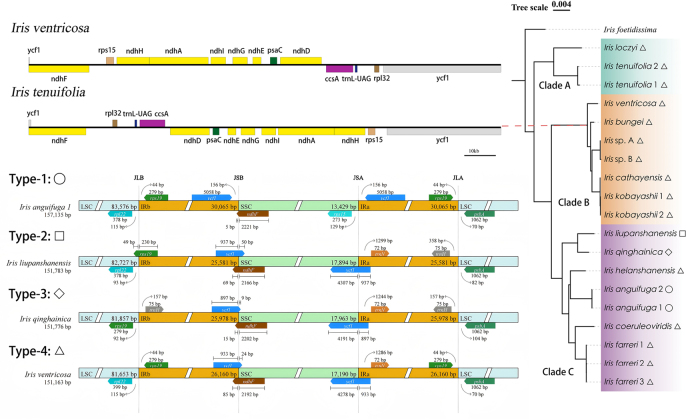
Phylogenetic relationships of the major clades in *I.* ser. *Tenuifoliae* inferred from plastome data, along with key structural evolutionary events (IR boundary types and inversions). The left panel illustrates the main types of plastome structures, with putative expansion/contraction events in the inverted repeat regions and inversion events marked in the figure.

Mauve alignment based on syntenic blocks revealed that gene order is highly conserved among *I.* ser. *Tenuifoliae* species (Fig. 2). However, a sequence inversion of approximately 17,000 bp was detected in the SSC region of 7 samples: *I. ventricosa*, *I. bungei*, *I.* sp. A, *I.* sp. B, *I. cathayensis*, *I. kobayashii* 1, and *I. kobayashii* 2. Inspection of the annotation results indicated that this inversion reverses 8 genes: *rps15*, *ndhH*, *ndhA*, *ndhI*, *ndhG*, *ndhE*, *psaC*, and *ndhD* (Fig. 3).

In the comparative analysis of IR boundary regions in the plastomes of the *I.* ser. *Tenuifoliae*, 4 distinct boundary types were identified based on the positions of the key genes *rps19* and *ycf1* relative to the IR/SC junctions. Type-1 exhibits the unique structure where both *rps19* and *ycf1* are entirely located within the IR region, a configuration unique to *I. anguifuga* (2 samples). Type-2 features *rps19* straddling the JLB junction and *ycf1* spanning the JSA/JSB junction, which was only found in *I. liupanshanensis*. Type-3 features *rps19* entirely located within the LSC region while *ycf1* spans the JSA/JSB junction, observed only in *I. qinghainica*. All remaining samples belong to Type-4, in which *rps19* is entirely located within the IR region and *ycf1* spans the JSA/JSB junction. Overall, the IR/SC boundary structures in the *I.* ser. *Tenuifoliae* species exhibit a high degree of commonality at the 4 junctions (JLB, JSB, JSA, JLA), although specific expansion or contraction events at the *rps19* and *ycf1* gene positions have occurred in some species. Using the majority state (Type-4) as the reference, Type-2 and Type-3 represent IR contraction, where *rps19* has shifted from inside the IR region outward, either spanning the JLB junction (Type-2) or moving entirely into LSC (Type-3). By contrast, Type-1 represents clear IR expansion, where *ycf1* has changed from spanning the junction to being entirely within the IR region, resulting in 2 complete copies. Complete IR boundary maps for all 19 samples are provided in Suppl. material 4.

### Phylogenetic analyses

**Figure 4.**
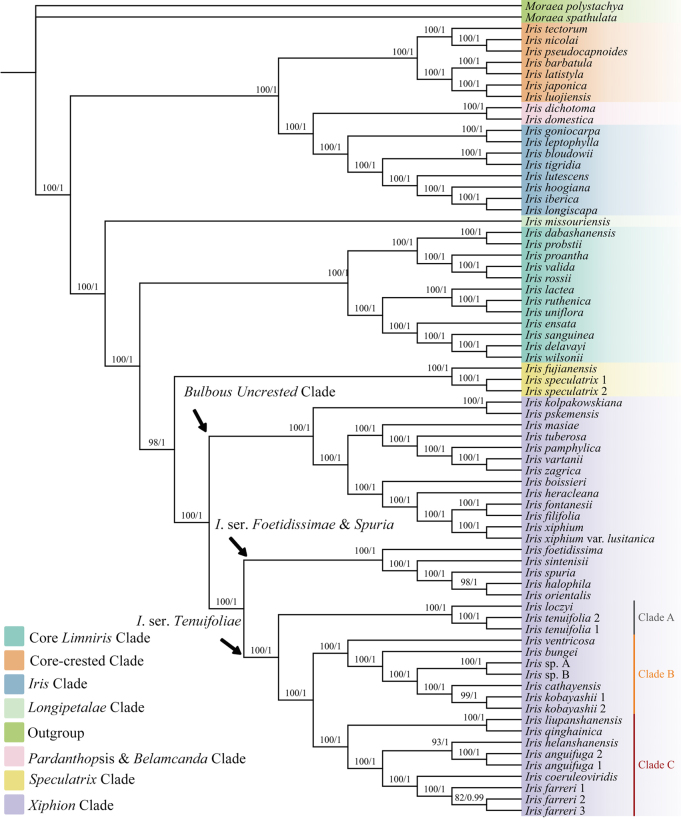
Molecular phylogenetic tree of *Iris* based on 70 plastid genomes (Combined maximum likelihood and Bayesian inference analysis). Nodal values indicate bootstrap percentages/Bayesian posterior probabilities.

The Maximum Likelihood (ML) and Bayesian Inference (BI) trees constructed using the chloroplast genome dataset showed congruent topologies (Fig. 4). The phylogenetic tree of *Iris* reconstructed in this study strongly supports its division into 7 monophyletic groups, which is consistent with the major clades defined in previous studies, such as the Core-crested Clade, Core *Limniris* Clade, and *Xiphion* Clade (Fig. 4). All key nodes defining these monophyletic groups received robust statistical support (Fig. 4). The Core-crested Clade + (*Pardanthopsis* & *Belamcanda* Clade + Bearded Clade) forms a well-supported clade, which is sister to another well-supported clade comprising *Longipetalae* Clade + (Core *Limniris* Clade + (*Speculatrix* Clade + *Xiphion* Clade))(BS = 100, PP = 1).

Within the *Xiphion* Clade, *I.* ser. *Tenuifoliae* and the clade (*I.* ser. *Foetidissimae* + *I.* ser. *Spuriae*) were recovered as sister groups (BS = 100, PP = 1). The clade comprising (*I.* ser. *Foetidissimae* + *I.* ser. *Spuriae*) + *I.* ser. *Tenuifoliae* is the sister group to the Bulbous Uncrested Clade (defined as all descendants of the last common ancestor possessing tunicate bulbs within the *Xiphion* Clade) (BS = 100, PP = 1). *I.* ser. *Tenuifoliae*, after incorporating 3 newly discovered taxa and 2 specimens requiring further study, was strongly supported as monophyletic in our results (BS = 100, PP = 1).

Furthermore, 3 highly supported branches were identified within *I.* ser. *Tenuifoliae*. *I. loczyi* and *I. tenuifolia* form Clade A, which is sister to the rest of *I.* ser. *Tenuifoliae* (BS = 100, PP = 1). Clade B in our analysis comprises 5 formally described taxa and 2 unidentified taxa. *I. ventricosa* and *I. bungei* represent successive early-diverging lineages within the clade, with both species exhibiting distinct aerial stems. The remaining species lacking aerial stems form a robust monophyletic group (BS = 100, PP = 1). The clade comprising *I.* sp. A and *I.* sp. B is sister to the clade comprising 2 samples of *I. kobayashii* and *I. cathayensis* (BS = 100, PP = 1). In Clade C, *I. qinghainica* and *I. liupanshanensis* form the earliest-diverging sister species pair within the clade (BS = 100, PP = 1). *I. helanshanensis* and the clade comprising 2 samples of *I. anguifuga* form a sister-group relationship (BS = 98, PP = 1), together constituting the second diverging lineage. Specimens of *I. farreri* from 3 localities form a monophyletic group, which is strongly supported as sister to *I. coeruleoviridis* (BS = 100, PP = 1).

## Discussion

### The phylogenetic position and delimitation of *I.* ser. *Tenuifoliae* within the genus *Iris*

In our phylogenetic results, the monophyly of the *Xiphion* Clade is strongly supported, consistent with the conventional molecular phylogenetic analysis (ML) by Mavrodiev et al. (2014) and Crespo et al. (2025). Similarly, a sister relationship between *I.* ser. *Tenuifoliae* and the clade (*I.* ser. *Foetidissimae* + *I.* ser. *Spuriae*) was recovered.

Species of *I.* ser. *Tenuifoliae*, as well as *I.* ser. *Foetidissimae* and *I.* ser. *Spuriae*, are predominantly rhizomatous perennial herbs. Their leaves are isobilateral, and in species with aerial stems, the flowering stem often shows structures identifiable as bract-like leaves. Similar structures are also present in other lineages of the *Xiphion* Clade, such as *I. grant-duffii* Baker, *I. filifolia* Boiss., and *I. xiphium* L., which may result from the reduction of multi-branched flowering stems. Additionally, many similarities are observed in floral morphology, such as pandurate to obovate sepals and erect petals (Mathew 1989). Their seeds usually lack appendages. (Zhao et al. 2000; Rodionenko 2005; Crespo 2011).

*I. anguifuga* has been clearly resolved as a member of *I.* ser. *Tenuifoliae*, showing close relationships with species such as *I. farreri* and *I. coeruleoviridis*. The flowering stem of *I. anguifuga* is distinctive, bearing a solitary flower with a single bract and 2 bract-like leaves on the stem. While Zhao (1980) and Rodionenko (2004) focused on the solitary bract, they did not investigate the bract-like leaves on the stem. In comparison, *I. coeruleoviridis* also produces solitary flowers but possesses 2 bracts and a single bract-like leaf on the stem. This suggests that the single bract in *I. anguifuga* may result from internode elongation between 2 bracts. In terms of growth habit, *I. anguifuga* is characterized by growth in autumn, winter, and spring, followed by withering and dormancy during summer (Zhao 1980). This pattern is likely an adaptation to the climatic conditions in its distribution area, where winters are relatively mild while summers are hot and rainy.

Dykes (1912) noted that *I. songarica* possesses a flowering stem structure similar to that of species in *I.* ser. *Spuriae*, while sharing a subterranean morphology comparable to species in *I.* ser. *Tenuifoliae*, considering it a connecting link between the 2 groups. In terms of floral morphology, the elongated ovary neck, narrowly triangular style crests, and prominently spotted perianth segments and style branches of *I. songarica* closely resemble those of *I. kobayashii* in *I.* ser. *Tenuifoliae* (Sennikov et al. 2023). In overall plant habit, it shares a similar clump-forming growth pattern and features straight, scarcely branched roots with other members of ser. *Tenuifoliae*. As samples of *I. songarica* were not available for this study, clarifying its phylogenetic position remains a key objective for future research.

### The distribution of *I.* ser. *Tenuifoliae*

Overall, the species of *I.* ser. *Tenuifoliae* are distributed across temperate regions from East Asia to Central Asia, with their distribution center located in the northeastern Qinghai-Tibet Plateau and adjacent areas. However, further analysis reveals that although the distribution ranges of the 3 identified clades are interdigitated, they are not entirely overlapping.

Clade A consists of *I. loczyi* and *I. tenuifolia*. *I. tenuifolia* inhabits stabilized sand dunes across Central Asia, Mongolia, northwestern China, and adjacent Russia, while *I. loczyi* is found in sunny alpine grasslands of the Tien Shan Mountains, Pamir Plateau, and Tibetan Plateau (Zhao et al. 2000; Sennikov et al. 2023). The 2 species exhibit some divergence in habitat preference, suggesting that ecological selection may have driven their differentiation. Zhao et al. (2000) noted that *I. loczyi*, identified from regions in China other than Xinjiang and western Tibet, might be related to *I. tenuifolia*. However, due to limited sampling, further studies are needed to clarify the relationship between *I. loczyi* and *I. tenuifolia*.

Clade B includes *I. ventricosa* and *I. bungei*, which possess distinct aerial stems, although the stem length varies with the depth of sand burial and sometimes does not emerge above the ground (Zhao 1985). It is worth noting that the presence or absence of aerial stems was considered an important taxonomic character by Rodionenko (1961). However, this distinguishing criterion was no longer applicable following the expansion of the series by Mathew (1989). In this study, 5 recognized species of *I.* ser. *Tenuifoliae* that lack aerial stems are distributed across 3 different clades. Within Clade B, the 2 groups lacking aerial stems show an east-west disjunction in distribution. *I.* sp. A and *I.* sp. B are distributed in the central-western regions of China (primarily in Shaanxi and Shanxi); these 2 taxa have not been previously recorded in the literature, nor are there any relevant collection records, and are therefore reported here as new records. *I. kobayashii* and *I. cathayensis* are found in central-eastern China, with the former occurring in Shandong and southern Liaoning, and the latter in Henan, Anhui, and Jiangsu. (Zhao et al. 2000). This suggests that the differentiation of the 2 lineages is likely the result of geographic isolation. Due to the inability to obtain detailed morphological data for *I.* sp. A and *I.* sp. B in this study, their relationship and status as independent species still require further in-depth research.

Clade C incorporates the 3 newly discovered species, resulting in a total of 6 species. *I. qinghainica* and *I. liupanshanensis* represent the earliest diverging sister species within this clade, with the former distributed in the northeastern Qinghai-Tibet Plateau and the latter occurring in the Liupan Mountain region (Zhao 1980). *I. helanshanensis* is found in the Helan Mountains and Luo Mountains, while *I. anguifuga* is distributed across mountainous areas of central-eastern China. It is noteworthy that *I. anguifuga* has relatively few collection records and is also encountered in cultivation; thus, its natural distribution range requires further investigation. *I. farreri* has a broad distribution, spanning the eastern and northeastern Qinghai-Tibet Plateau as well as the Liupan Mountains. *I. coeruleoviridis* is currently known only from a relatively limited area on the western section of the Qinling Mountains. The distinct pattern of geographic isolation observed among sister species within this clade underscores the pivotal role of the complex East Asian mountain system in the process of speciation. Geographic barriers have not only restricted gene flow between populations but also promoted adaptive evolution in independent settings, thereby shaping the species diversity of this group.

### Structural variations and phylogenetic signals: inversion and dynamic evolution of the IR region

Large-scale structural variations in chloroplast genomes, particularly inversions, have been widely recognized as powerful phylogenetic signals due to their rarity and stable inheritance. For example, in *Oenothera* L., a ~56 kb inversion has been identified as a synapomorphy of *O.* subsect. *Oenothera* (Greiner et al. 2008). Similarly, the conserved inversion located in the SSC region discovered in Clade B of *I.* ser. *Tenuifoliae* in this study also constitutes a unique genomic structural marker for this clade, providing decisive evidence independent of sequence data for its monophyly.

At the same time, our study reveals that expansion and contraction of IR boundaries are largely conserved among different species of *I.* ser. *Tenuifoliae*, with only *I. liupanshanensis* and *I. qinghainica* showing varying degrees of contraction, and *I. anguifuga* exhibiting a more substantial expansion. The non-conservative changes (contraction or expansion) of IR boundaries observed in a few species suggest that this region remains dynamically active during localized evolution. Such variation may affect the copy number and integrity of boundary genes (e.g., *rps19* and *ycf1*), potentially exerting evolutionary or functional influences on the species. Future studies could expand sampling across different ecotypes within the genus *Iris* to explore whether these genomic structural variations are associated with specific environmental adaptations.

## Taxonomic treatment

### Iris liupanshanensis

Taxon classificationPlantaeAsparagalesIridaceae

H.T.Li, Z.Z.Zhang & T.Y.Zuo sp. nov.

E797F5BF-C85D-5BDA-BC32-1CF6A89D7AEC

urn:lsid:ipni.org:names:77380911-1

Fig. 5

#### Diagnosis.

This new species is morphologically similar to *I. ventricosa*, but differs by its broader leaves (10–12 mm vs. 3–4 mm), longer and narrower bracts (10–13 × 0.9–1.5 cm vs. 6–8 × 2.5–4 cm), and larger flowers in diameter (8–9 cm vs. 6–7 cm).

**Figure 5.**
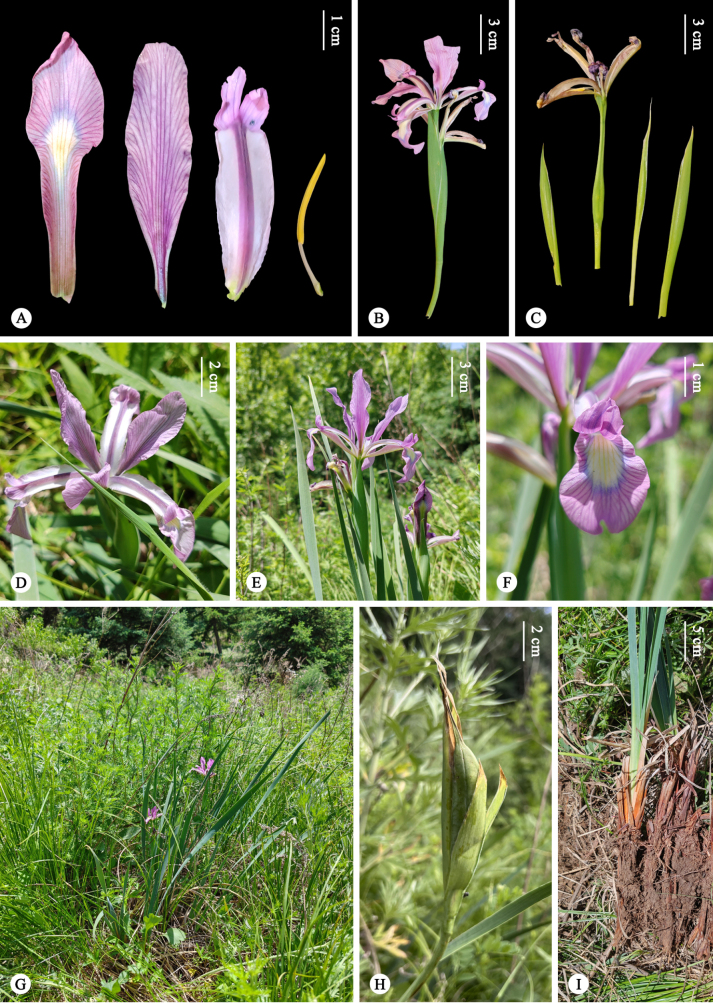
*Iris liupanshanensis* H.T.Li, Z.Z.Zhang & T.Y.Zuo, sp. nov. **A**. Sepals, petals, style branches and stamens; **B**. Inflorescence; **C**. Bracts and ovary; **D–F**. Flower; **G**. Plant; **H**. Fruit; **I**. Persistent leaf sheath and roots.

#### Type.

China • Ningxia, Guyuan City, Yuanzhou District, Gujiang Road, on the grassland of the hillside; 2085 m; 35°57.1140'N, 106°8.1287'E; 17 June 2025 (fl); *Z.Z.Zhang ZZZ-GJL-001* (holotype: NAS!, isotype: SG!).

#### Description.

Perennial caespitose herbs with persistent old leaf bases; sheaths subterranean, dark brown, rigid. ***Roots*** tough, straight descending, unbranched. ***Leaves*** linear, grey-green, 55–75 cm × 10–12 mm, with ca. 10 inconspicuous veins. ***Flowering stems*** 25–40 cm tall, terete, with a single sheathing leaf at the base. ***Bracts*** 3, herbaceous, green, margin membranous, broadly lanceolate, 10–13 × 0.9–1.5 cm, 2-flowered. ***Flowers*** deep pink, 8–9 cm in diam.; pedicel 3–7 cm long; perianth tube 0.5–0.7 cm long; sepals spreading, pandurate, 5–6.5 × 0.8–1 cm, limb reflexed downward, central part of limb cream to pale yellow, velvety, not smooth; petals erect, oblanceolate, 5–6.3 × 1.5–2.0 cm; stamens ca. 3.4 cm long, filament ca. 8 mm long, anthers yellow, ca. 2.6 cm long; style branches slightly curved-arching, ridge deep pink, both sides white, margin membranous, ca. 4.5 × 1.5 cm, apex lobed, reflexed upward, lobes linear-broadly triangular, ca. 1.4 cm × 5 mm; stigmatic lip bilobed; ovary cylindric, 4.5–5.5 cm long, with a neck 2.5–3.1 cm long. ***Capsule*** oblong-ovoid, 5–7 × ca. 2 cm, base rounded, apex long-acuminate, beak 2–3 cm long. ***Seeds*** 4–5 mm, irregularly globose; testa wrinkled. Flowering June, fruiting July–August.

#### Distribution and ecology.

**Figure 6.**
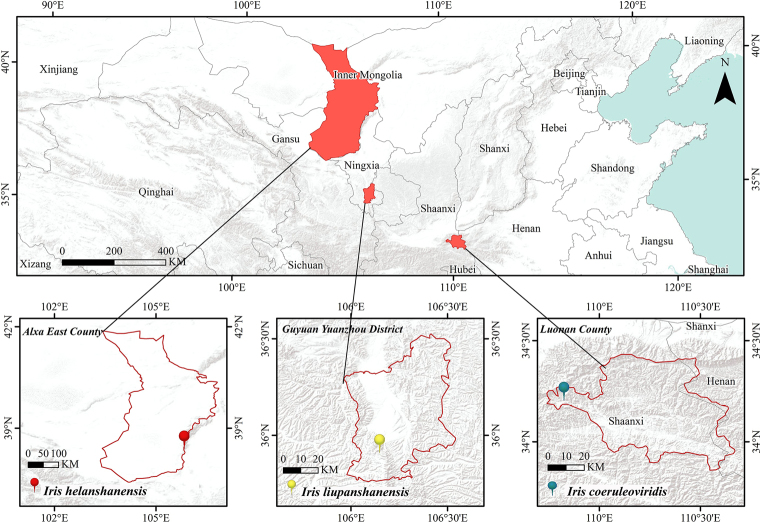
The known distribution of *Iris liupanshanensis*, *I. helanshanensis* and *I. coeruleoviridis* in China.

*I. liupanshanensis* is distributed in the Liupan Mountains region (spanning the eastern part of Gansu Province and the southern part of the Ningxia Hui Autonomous Region, China), growing in open or semi-open montane grasslands (Fig. 6).

#### Etymology.

The specific epithet “*liupanshanensis*” is derived from the type locality of the species, which is located in the northern section of the Liupan Mountains.

#### Preliminary conservation status.

According to the IUCN (2024) Red List Categories and Criteria, *I. liupanshanensis* is preliminarily assessed as Data Deficient (DD) due to currently insufficient information. Its overall distribution range, population size, and specific threats are currently unknown. Targeted field surveys are therefore urgently required to collect data on population dynamics and habitat status, which would enable a more accurate future assessment.

### Iris helanshanensis

Taxon classificationPlantaeAsparagalesIridaceae

Z.Z.Zhang, T.Y.Zuo & H.T.Li sp. nov.

10CB19E4-2169-5911-BA53-6E932BEBE342

urn:lsid:ipni.org:names:77380912-1

Fig. 7

#### Diagnosis.

This new species is morphologically similar to *I. farreri*, but differs by its shorter bracts (6.4–7 cm vs. 7–14 cm), ovary lacking elongated necks, and beakless fruits.

**Figure 7.**
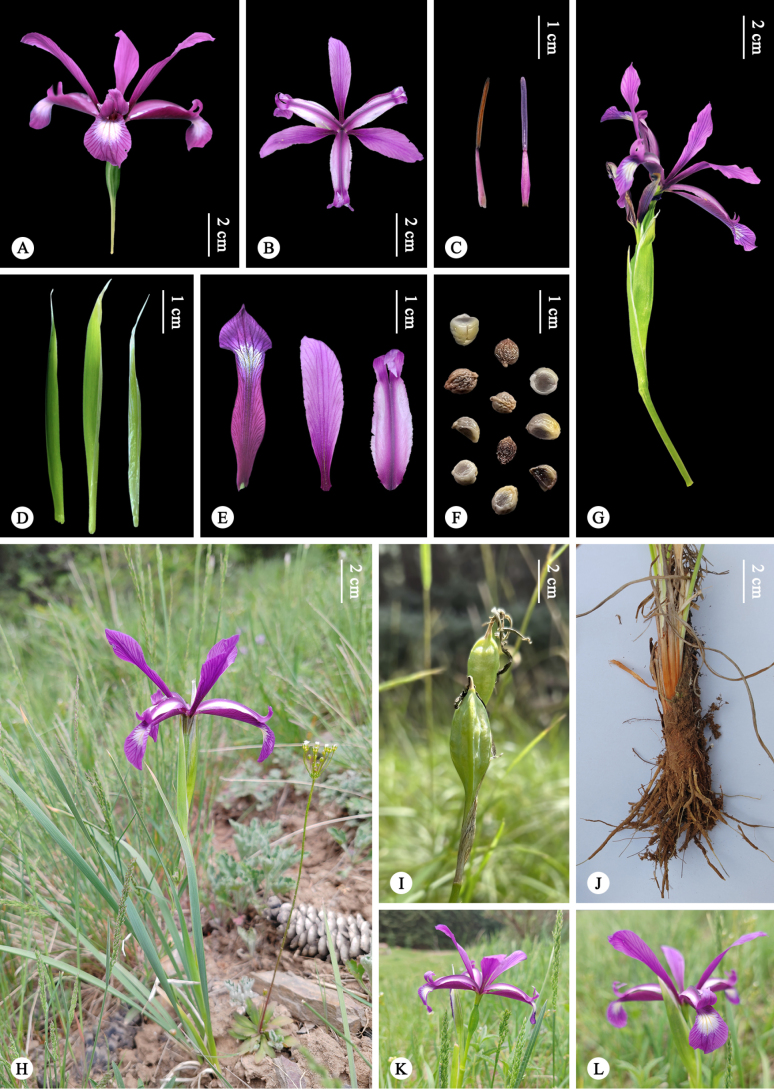
*Iris helanshanensis* Z.Z.Zhang, T.Y.Zuo & H.T.Li, sp. nov. **A**, **B**. Flower; **C**. Stamens; **D**. Bracts; **E**. Sepals, petals and style branches; **F**. Seeds; **G**. Inflorescence; **H**. Plant; **I**. Fruit; **J**. Persistent leaf sheath and roots; **K, L**. Flower.

#### Type.

China • Inner Mongolia Autonomous Region, Alxa League, Nansi (Guangzong Temple) Tourist Area, on the open grassland in the valley; 2395 m; 38°39.4295'N, 105°50.2786'E; 18 June 2025 (fl); *Z.Z.Zhang ZZZ-NS-001* (holotype: NAS!).

#### Description.

Perennial caespitose herb. ***Plant base*** surrounded by fibrous remnants of old brown leaf sheaths. ***Rhizomes*** knobbly, woody. ***Roots*** tough, unbranched, nearly uniform in diameter throughout. ***Leaves*** grey-green, linear, 18–35 cm × 4–6 mm, with 3–5 longitudinal veins. ***Flowering stems*** 15–40 cm tall, smooth, bearing a single bract-like leaf near the middle. ***Bracts*** 3, herbaceous, green, 6.4–7 × 0.5–1 cm, apex shortly acuminate, 2-flowered. ***Flowers*** violet to purple, 7.5–8 cm in diam.; pedicel 2.3–3 cm long; perianth tube ca. 9 mm long; sepals spreading, pandurate, 4–4.5 × ca. 1 cm, limb reflexed downward, claw white or pale yellow with purple reticulate veins; petals erect, oblanceolate, 4.2–4.5 × ca. 1 cm; stamens ca. 2.8 cm long, anthers dark purple; style branches slightly curved, 4–4.5 × ca. 1 cm, apex lobes narrowly triangular; stigmatic lip bilobed. ***Ovary*** trigonous-ovoid, 1.4–2 cm long. ***Capsule*** trigonous-ovoid, 3.5–4.5(–5.5) × 1.2–2 cm, pericarp membranous, prominently reticulate-veined. ***Seeds*** brown, irregularly polyhedral, without appendages; testa wrinkled. Flowering May–June, fruiting July–September.

#### Distribution and ecology.

*I. helanshanensis* is distributed in the Helan Mountains and Luo Mountains (Inner Mongolia Autonomous Region and Ningxia Hui Autonomous Region, China), where it grows in open grassy slopes or forest edges (Fig. 6).

#### Etymology.

The type locality is situated in the Helan Mountains, hence the specific epithet “*helanshanensis*” is chosen.

#### Preliminary conservation status.

Based on the IUCN (2024) Red List Categories and Criteria (Version 3.1, 2012), *I. helanshanensis* is preliminarily assessed as Vulnerable (VU). This assessment is based on its simultaneous fulfilment of the following 2 criteria: D1 (total number of mature individuals estimated to be fewer than 1,000) and D2 (Area of Occupancy is estimated to be less than 20 km^2^, coupled with a very limited number of locations). Plausible future threats include habitat degradation and fragmentation resulting from ongoing tourism development, such as trail construction, visitor foot traffic, and associated infrastructure expansion, which may negatively impact the long-term survival of this taxon. It is strongly recommended that systematic field surveys be conducted promptly to verify its distribution range, population dynamics, and specific threats, thereby providing a scientific basis for confirming its final conservation status.

#### Notes.

The distribution of this species in the Luo Mountains, at the border between Hongsibu District and Tongxin County in Wuzhong City, Ningxia Hui Autonomous Region, is only documented through a single herbarium specimen (BNU0019183). Since the Luo Mountains has been designated as a national nature reserve, further verification is required to assess the species’ population status within this region.

#### Other specimens examined.

1. China • Ningxia Hui Autonomous Region, Wuzhong City, Tongxin County, Luoshan Nature Reserve, on grassy mountain slopes. 1807 m, 26 June 2015 (fr), *BNU Expedition LS2015023*, BNU0019183 3. China • Inner Mongolia Autonomous Region, Alxa League, Alxa East County, Bayan Hot Town, on the way from South Temple to Bayan Sunbu’er, subalpine meadow, 2585 m, 20 June 2021 (fl), *Bing Liu, Yalei Feng & Xinxin Zhou 12481*, PE 02430836, PE 02430837, PE 02442401.

### Iris coeruleoviridis

Taxon classificationPlantaeAsparagalesIridaceae

T.Y.Zuo, H.T.Li & Z.Z.Zhang sp. nov.

6E6445D7-A8EA-5FB0-A243-DA7478C9444F

urn:lsid:ipni.org:names:77380913-1

Fig. 8

#### Diagnosis.

This new species is morphologically similar to *I. anguifuga*, but differs by its broader leaves [7–10(–12) mm vs. 5–7 mm], smaller flowers in diameter (6–6.5 cm vs. ca. 10 cm).

**Figure 8.**
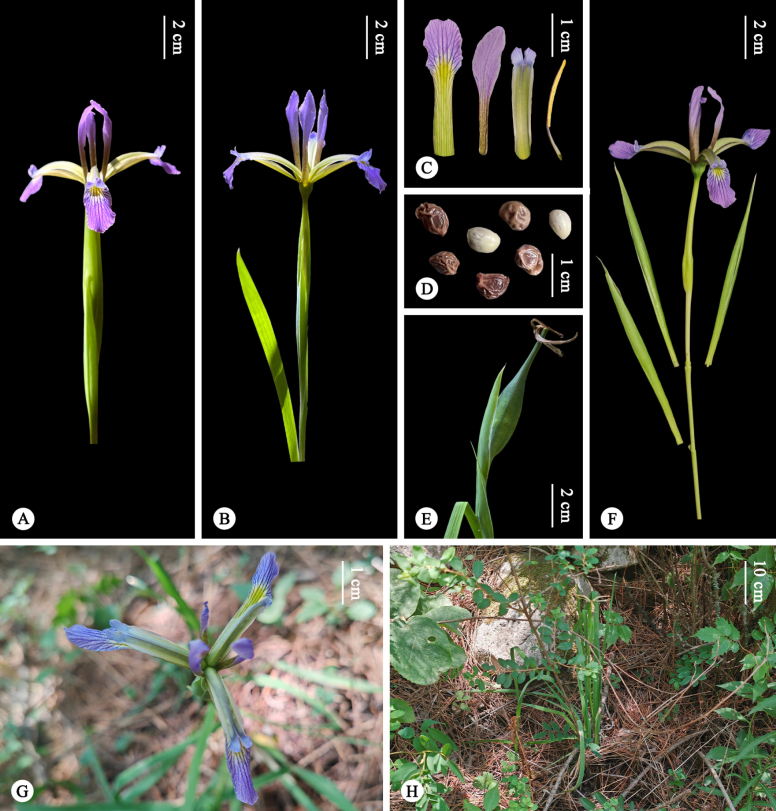
*Iris coeruleoviridis* T.Y.Zuo, H.T.Li & Z.Z.Zhang, sp. nov. **A**, **B**. Inflorescence; **C**. Sepals, petals, style branches and stamens; **D**. Seeds; **E**. Fruit; **F**. Bracts and ovary; **G**. Flower; **H**. Plant.

#### Type.

China • Shaanxi Province, Luonan County, Luoyuan Town, near the headwaters of the Luo River in the Caolianling area; 1566 m; 34°15.0736'N, 109°49.4950'E; 16 June 2025 (fl); *Z.Z.Zhang ZZZ-CLL-001* (holotype: NAS!, isotypes: SG!, CSH!).

#### Description.

Perennial herb, base surrounded by sheaths and fibers. ***Rhizome*** thick, irregularly lumpy. ***Leaves*** linear, 45–65 cm × 7–10(–12) mm, with 3–6 veins. ***Flowering stem*** 40–55 cm tall, with 2–3 cauline leaves borne at the base and 1 bract-like leaf inserted near the middle. ***Bracts*** 2, narrowly lanceolate to linear-lanceolate, 6–9.5 cm long, apex acuminate, 1-flowered. ***Flowers*** faintly fragrant, 6–6.5 cm in diam.; pedicel 2–3 cm long; perianth tube ca. 0.5 cm long; sepals spreading, oblanceolate, ca. 3.5 cm × 7–10 mm, limb apex bluish purple, claw yellowish green; petals erect, oblanceolate, 2.8–3.1 cm × 5–7 mm, upper part bluish purple, lower part brown, densely spotted; stamens 2.2–2.4 cm long; anthers bright yellow, 1.4–1.6 cm long, filaments flattened; style branches arched, margins membranous, ca. 3 cm × 5 mm, apex lobes triangular, ca. 5 × 3 mm; stigmatic lip bilobed; ovary trigonous, ca. 3.6 cm long, ca. 4 mm in diam., with a neck 2.3–3.5 cm long. ***Capsule*** trigonous-fusiform, 6.5–8 × ca. 1.5 cm, apex with a long beak ca. 2.7 cm long, fruiting pedicel 2.5–3.5 cm long. ***Seeds*** subglobose, without appendages, 4–5 mm in diam; testa wrinkled. Flowering June–July, fruiting August–September.

#### Distribution and ecology.

This new species is currently known only from the Caolianling area in the western Qinling Mountains, where it grows in forest understory and along forest margins. Its full distribution range requires further investigation (Fig. 6).

#### Etymology.

Since this new species possesses a style that is green with a bluish tinge at the apex, the specific epithet “*coeruleoviridis*” was chosen.

#### Preliminary conservation status.

Based on the preliminary assessment using the IUCN (2024) Red List Categories and Criteria, *I. coeruleoviridis* is assigned a threat category of Data Deficient (DD). The species is currently known only from the Caolianling area in the western Qinling Mountains. The available information is insufficient to fully assess its extinction risk (e.g., due to possibly undiscovered populations or a lack of data on population dynamics and threat factors), and it is therefore classified as Data Deficient.

##### Key to *Iris* ser. *Tenuifoliae*

**Table:** 

1	Flowering stem elongated, clearly above ground.	**2**
–	Flowering stem short, not emerging above ground	**9**
2	Rhizomes distinctly swollen, entirely block-like	**3**
–	Rhizomes indistinctly swollen, knobbly	**4**
3	Spathes 10–13.5 cm; summer dormant	***I. anguifuga***
–	Spathes 6–9.5 cm; winter dormant	***I. coeruleoviridis***
4	Ovary neck > 2.5 cm.	**5**
–	Ovary neck < 2.5 cm or absent	**7**
5	Flowering stem > 30 cm	***I. songarica***
–	Flowering stem < 30 cm	**6**
6	Spathes without transverse veins	***I. bungei***
–	Spathes with transverse veins	***I. ventricosa***
7	Style branches with purple-brown spots	***I. farreri***
–	Style branches without purple-brown spots.	**8**
8	Leaves 10–12 mm wide	***I. liupanshanensis***
–	Leaves 4–6 mm wide	***I. helanshanensis***
9	Leaves filiform, 1.5–2 mm wide	***I. tenuifolia***
–	Leaves linear, more than 2 mm wide	**10**
10	Ovary neck > 10 cm	***I. loczyi***
–	Ovary neck < 10 cm	**11**
11	Flowers yellow with purple spots	***I. kobayashii***
–	Flowers bluish violet	**12**
12	Flowers 6–7.5 cm in diam; leaves 3–4(6) mm wide	***I. cathayensis***
–	Flowers 4.5–5 cm in diam; leaves 2–3 mm wide	***I. qinghainica***

## Supplementary Material

XML Treatment for Iris liupanshanensis

XML Treatment for Iris helanshanensis

XML Treatment for Iris coeruleoviridis

## Additional information

### Artificial Intelligence (AI) use

The authors accept full responsibility for the content of the manuscript, including the disclosure of any use of AI.

No AI tools were used in the preparation of this manuscript.

### Author ORCIDs

Z. Zhang https://orcid.org/0009-0000-0873-6440

H. Li https://orcid.org/0009-0008-5638-9445

G. Zhang https://orcid.org/0000-0002-6360-5109

T. Zuo https://orcid.org/0009-0007-7456-5724

### Data availability

All of the data that support the findings of this study are available in the main text or Supplementary Information.

## Supplementary materials

Supplementary material 1 Occurrences Data type xlsx Explanation note Examined specimens of *Iris* ser. *Tenuifoliae*. https://binary.pensoft.net/file/1666536This dataset is made available under the Open Database License (http://opendatacommons.org/licenses/odbl/1.0/). The Open Database License (ODbL) is a license agreement intended to allow users to freely share, modify, and use this Dataset while maintaining this same freedom for others, provided that the original source and author(s) are credited. Zhongzheng Zhang

Supplementary material 2 Accession numbers Data type xlsx Explanation note Samplings and their information used in this study. https://binary.pensoft.net/file/1666537This dataset is made available under the Open Database License (http://opendatacommons.org/licenses/odbl/1.0/). The Open Database License (ODbL) is a license agreement intended to allow users to freely share, modify, and use this Dataset while maintaining this same freedom for others, provided that the original source and author(s) are credited. Zhongzheng Zhang

Supplementary material 3 Genomic data Data type xlsx Explanation note Features of plastid genomes in 20 *Iris* samples. https://binary.pensoft.net/file/1666538This dataset is made available under the Open Database License (http://opendatacommons.org/licenses/odbl/1.0/). The Open Database License (ODbL) is a license agreement intended to allow users to freely share, modify, and use this Dataset while maintaining this same freedom for others, provided that the original source and author(s) are credited. Zhongzheng Zhang

Supplementary material 4 Images Data type jpg Explanation note Comparison of the junction regions (LSC,SSC,IR) across 18 *Iris* ser. *Tenuifoliae* species. https://binary.pensoft.net/file/1666539This dataset is made available under the Open Database License (http://opendatacommons.org/licenses/odbl/1.0/). The Open Database License (ODbL) is a license agreement intended to allow users to freely share, modify, and use this Dataset while maintaining this same freedom for others, provided that the original source and author(s) are credited. Zhongzheng Zhang
