# Supplementary figures and images for: Phylogeny and taxonomy of Iris (Iridaceae) series Tenuifoliae: Discovery of a clade with chloroplast genome inversion and description of three new species
Source: PhytoKeys. 2026 Jun 2;275:299–320. doi: 10.3897/phytokeys.275.184036 (PMC13250613; doi:10.3897/phytokeys.275.184036)

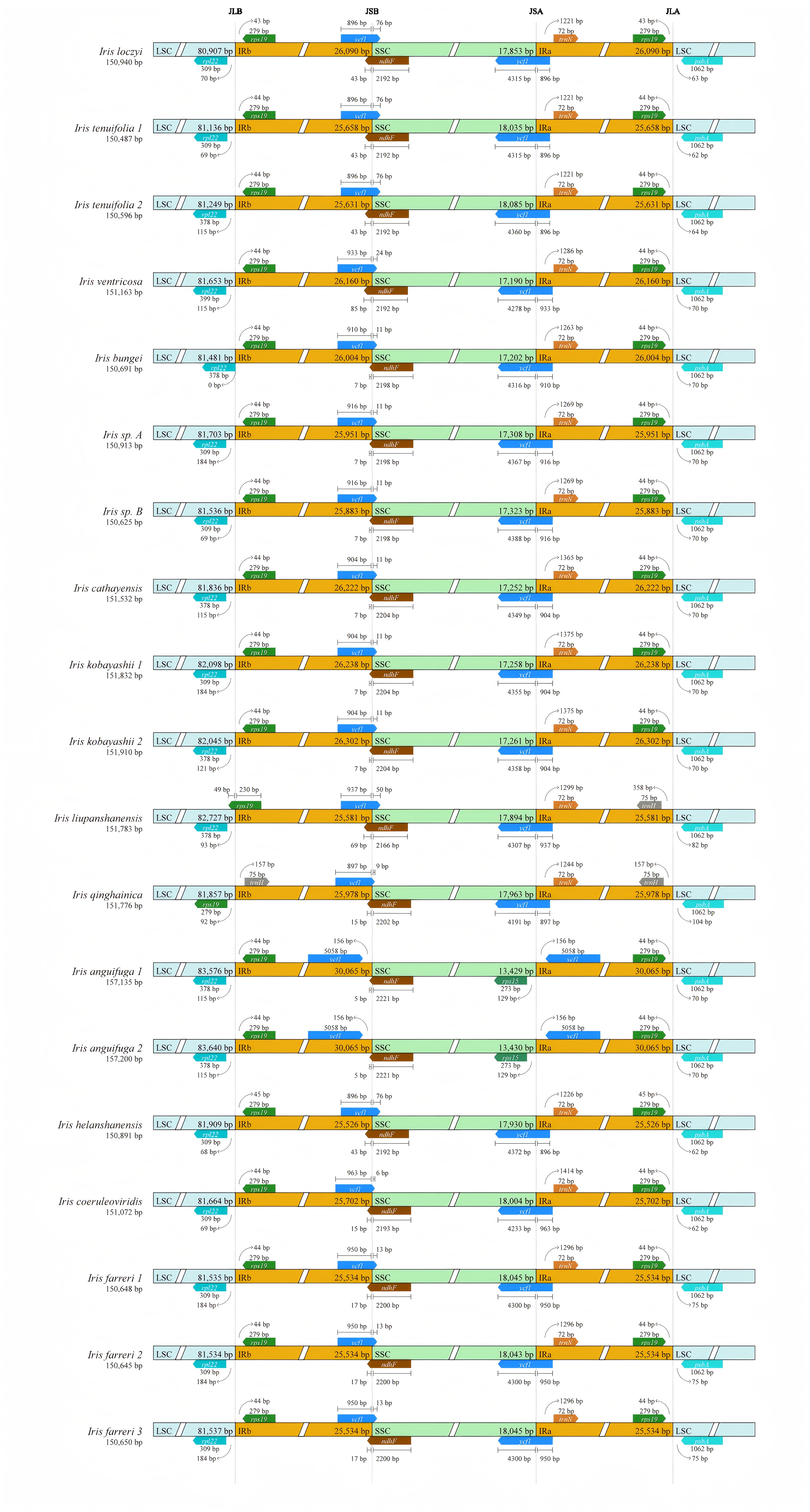

Supplement: Supplementary material 4 — Images [file phytokeys-275-299_article-184036__-s004.jpg]
